# Prediabetes Is Associated with Increased Prevalence of Sleep-Disordered Breathing

**DOI:** 10.3390/jcm11051413

**Published:** 2022-03-04

**Authors:** Enric Sánchez, Esther Sapiña-Beltrán, Ricard Gavaldà, Ferran Barbé, Gerard Torres, Ariadna Sauret, Mireia Dalmases, Carolina López-Cano, Liliana Gutiérrez-Carrasquilla, Marcelino Bermúdez-López, Elvira Fernández, Francisco Purroy, Eva Castro-Boqué, Cristina Farràs-Sallés, Reinald Pamplona, Dídac Mauricio, Cristina Hernández, Rafael Simó, Albert Lecube, on behalf of the ILERVAS Project Collaborators

**Affiliations:** 1 Endocrinology and Nutrition Department, University Hospital Arnau de Vilanova, 25198 Lleida, Spain; esanchez@irblleida.cat (E.S.); ariadnags973@gmail.com (A.S.); karolopezc@gmail.com (C.L.-C.); liligutierrezc@gmail.com (L.G.-C.); 2 Obesity, Diabetes and Metabolism (ODIM) Research Group, Institut de Recerca Biomèdica de Lleida (IRBLleida), 25198 Lleida, Spain; 3 University of Lleida, 25003 Lleida, Spain; esther.sb91@gmail.com (E.S.-B.); febarbe.lleida.ics@gencat.cat (F.B.); gtorres@gss.scs.es (G.T.); mdalmases.lleida.ics@gencat.cat (M.D.); fpurroygarcia@gmail.com (F.P.); reinald.pamplona@mex.udl.cat (R.P.); 4 Respiratory Department, University Hospital Arnau de Vilanova and Santa María, 25198 Lleida, Spain; 5 Group of Translational Research in Respiratory Medicine, Institut de Recerca Biomèdica de Lleida (IRBLleida), 25198 Lleida, Spain; 6 Centro de Investigación Biomédica en Red de Enfermedades Respiratorias (CIBERES), Instituto de Salud Carlos III (ISCIII), 28029 Madrid, Spain; 7 Amalfi Analytics, Polytechnic University of Catalonia, 08034 Barcelona, Spain; rgavalda@irblleida.cat; 8 Vascular and Renal Translational Research Group, Institut de Recerca Biomèdica de Lleida (IRBLleida), 25198 Lleida, Spain; mbermudez@irblleida.cat (M.B.-L.); efernandez@irblleida.cat (E.F.); ecastro@irblleida.cat (E.C.-B.); 9 Red de Investigación Renal, Instituto de Salud Carlos III (RedinRen-ISCIII), 28029 Madrid, Spain; 10 Stroke Unit, University Hospital Arnau de Vilanova, 25198 Lleida, Spain; 11 Clinical Neurosciences Group, Institut de Recerca Biomèdica de Lleida (IRBLleida), 25198 Lleida, Spain; 12 Applied Epidemiology Research Group, Institut de Recerca Biomèdica de Lleida (IRBLleida), 25198 Lleida, Spain; cfarras.lleida.ics@gencat.cat; 13 Lleida Research Support Unit, Jordi Gol i Gurina University Institute for Research in Primary Health Care (IDIAPJGol), 25007 Lleida, Spain; 14 Department of Experimental Medicine, Institut de Recerca Biomèdica de Lleida (IRBLleida), 25198 Lleida, Spain; 15 Department of Endocrinology and Nutrition, Hospital de la Santa Creu i Sant Pau, 08041 Barcelona, Spain; didacmauricio@gmail.com; 16 Sant Pau Biomedical Research Institute (IIB Sant Pau), 08041 Barcelona, Spain; 17 Centro de Investigación Biomédica en Red de Diabetes y Enfermedades Metabólicas Asociadas (CIBERDEM), Instituto de Salud Carlos III (ISCIII), 28029 Madrid, Spain; cristina.hernandez@vhir.org; 18 Grupo de Investigación Epidemiológica en Diabetes des de Atención Primaria (DAP_Cat Group), Unitat de Suport a la Recerca de Barcelona, Jordi Gol i Gurina University Institute for Research in Primary Health Care (IDIAPJGol), 08007 Barcelona, Spain; 19 Endocrinology and Nutrition Department, University Hospital Vall d’Hebron, 08024 Barcelona, Spain; 20 Diabetes and Metabolism Research Unit, Vall d’Hebron Institut de Recerca (VHIR), 08024 Barcelona, Spain; 21 Autonomous University of Barcelona, 08024 Barcelona, Spain

**Keywords:** apnea, glycated hemoglobin, hypopnea, prediabetes, obstructive sleep apnea

## Abstract

Type 2 diabetes leads to severe nocturnal hypoxemia, with an increase in apnea events and daytime sleepiness. Hence, we assessed sleep breathing parameters in the prediabetes stage. A cross-sectional study conducted on 966 middle-aged subjects without known pulmonary disease (311 patients with prediabetes and 655 controls with normal glucose metabolism) was conducted. Prediabetes was defined by glycated hemoglobin (HbA1c), and a nonattended overnight home sleep study was performed. Participants with prediabetes (*n* = 311) displayed a higher apnea–hypopnea index (AHI: 12.7 (6.1;24.3) vs. 9.5 (4.2;19.6) events/h, *p* < 0.001) and hypopnea index (HI: 8.4 (4.0;14.9) vs. 6.0 (2.7;12.6) events/h, *p* < 0.001) than controls, without differences in the apnea index. Altogether, the prevalence of obstructive sleep apnea was higher in subjects with prediabetes than in controls (78.1 vs. 69.9%, *p* = 0.007). Additionally, subjects with prediabetes presented impaired measurements of the median and minimum nocturnal oxygen saturation, the percentage of time spent with oxygen saturations below 90%, and the 4% oxygen desaturation index in comparison with individuals without prediabetes (*p* < 0.001 for all). After adjusting for age, sex, and the presence of obesity, HbA1c correlated with the HI in the entire population (r = 0.141, *p* < 0.001), and the presence of prediabetes was independently associated with the AHI (B = 2.20 (0.10 to 4.31), *p* = 0.040) as well as the HI (B = 1.87 (0.61 to 3.14), *p* = 0.004) in the multiple linear regression model. We conclude that prediabetes is an independent risk factor for an increased AHI after adjusting for age, sex, and obesity. The enhanced AHI is mainly associated with increments in the hypopnea events.

## 1. Introduction

In recent years, there has been growing evidence suggesting that type 2 diabetes can lead to the development of sleep breathing disorders (SBD) [1]. The deleterious effect of type 2 diabetes on nocturnal sleep breathing includes increased nocturnal awakenings, higher sleep fragmentation through higher rates of microarousals, changes in sleep architecture, sleep quality reduction and, consequently, excessive daytime sleepiness [2,3,4]. Furthermore, type 2 diabetes appears to be an independent risk factor for severe hypoxemia [5]. The Sweet Sleep study characterized obstructive sleep apnea (OSA) in patients with type 2 diabetes, providing evidence that the composition of their apnea–hypopnea index (AHI) is characterized by an increase in apnea events, with no differences or even reduction in hypopnea episodes [2]. Additionally, a small interventional study with 35 patients with type 2 diabetes and OSA has recently shown how the improvement of glycemic control without significant weight loss exerts beneficial effects on sleep breathing parameters [6]. This multifaceted relationship between diabetes milieu and sleep breathing is based on several pathophysiological mechanisms that include insulin resistance, inflammatory and oxidative stress-activated signaling pathways, leptin resistance and abnormalities in the autonomic nervous system [1,7]. A recent analysis of 151,194 participants from three prospective U.S. cohorts has showed that individuals with insulin-treated diabetes had 43% higher OSA risk when compared to those without diabetes [8]. These data possibly indicate that SBD appears in established diabetes with the worst metabolic control, which requires intensive glucose lowering.

Little information exists regarding the potential nighttime respiratory dysfunction in subjects with prediabetes. This prodromal stage in the hyperglycemia continuum that exists before the clinical diagnosis of type 2 diabetes affects 34.5% of all United States adults based on their fasting glucose or glycated hemoglobin (HbA1c) level [9]. Some of the etiopathogenetic mechanisms involved in the development of SDB in type 2 diabetes, such as insulin resistance and low-grade inflammation, are also part of the prediabetes environment. In fact, data from the National Health and Nutrition Examination Survey 2005–2008 showed that self-reported markers of SDB (sleep duration, snoring, snorting, and daytime sleepiness) were associated with prediabetes [10].

On this basis, our main goal was to test the impact of prediabetes in the sleep breathing pattern in a cross-sectional study of 989 middle-aged subjects without type 2 diabetes.

## 2. Materials and Methods

### 2.1. Ethics Approval

The protocol was approved by the Arnau de Vilanova University Hospital Ethics Committee (CEIC-1410). Moreover, the trial was conducted according to the ethical guidelines of the Helsinki Declaration and Spanish legislation regarding the protection of personal information was also followed. Written informed consent was provided by all individuals when they were included in the study. Informed consent was obtained from all individual participants included in the study.

### 2.2. Design of the Study and Report of the Study Individuals

The ILERVAS project is an ongoing randomized intervention study to assess the prevalence of subclinical vascular disease in the province of Lleida, Spain (ClinTrials.gov Identifier: NCT03228459, 25 July 2017) [11,12]. A total of 8330 middle-aged participants were recruited from diverse primary health care centers between January 2015 and December 2018. The inclusion criteria were women aged between 50 and 70, men aged between 45 and 65, and the presence of at least one cardiovascular risk factor (dyslipidemia, hypertension, obesity, smoking and/or having a first-degree relative with premature cardiovascular disease). The exclusion criteria were medical history of cardiovascular disease, type 2 diabetes, chronic kidney disease, active neoplasia, a life expectancy less than 18 months, institutionalized population (jail and penitentiary inmates, patients at psychiatric hospitals, persons in nursing homes, and persons in boarding schools) and pregnancy.

For the present study, 2411 consecutive subjects were assessed for eligibility between March 2017 and September 2018 and invited to perform a nonattended cardiorespiratory polygraphy. We excluded 1335 for the following reasons: unwillingness to participate in the study (*n* = 932), unable to locate by telephone (*n* = 285), patients under treatment with continuous positive airway pressure (CPAP) (*n* = 81), previously undiagnosed type 2 diabetes (*n* = 15), and unknown kidney disease (*n* = 22). Additionally, 110 subjects with a first unsatisfactory sleep study refused to repeat it a second time. Therefore, the investigation was finally performed with nine hundred and sixty-six individuals: 311 patients with prediabetes and 655 controls with normal glucose metabolism (Supplemental Figure S1).

### 2.3. Diagnosis of Prediabetes

Following the present American Diabetes Association guidelines, prediabetes was defined as an HbA1c between 39 and 47 mmol/mol (5.7 to 6.4%) and a normal glucose metabolism as an HbA1c < 39 mmol/mol (<5.7%) [13]. The HbA1c test was conducted on capillary blood using a point-of-care device (Cobas B 101^®^, Roche Diagnostics S.L., Sant Cugat del Vallès, Spain), based on a latex agglutination inhibition immunoassay technique that meets the generally accepted performance standards for HbA1c [14].

### 2.4. Nighttime Respiratory Function Assessment

All participants underwent a nonattended overnight home sleep study using a cardiorespiratory polygraphy (ApneaLink™ device, Resmed, Sydney, Australia) according to standard techniques [15]. Oronasal flow, thoracoabdominal movements and pulse oximetry were recorded. Cardiorespiratory polygraph records were scored manually according to standard criteria, and records with less than 3 h of sleep time were repeated. Apnea was defined as an absence of or reduction in nasal airflow of >90% with a duration of at least 10 s. A hypopnea was defined as a reduction of 30% to 90% in oronasal airflow for at least 10 s and associated with a drop in arterial oxygen saturation (SpO2) of at least 3.0%. The AHI was defined as the sum of apneas plus hypopneas recorded during the study per h of monitoring time, and participants were classified into non-OSA (AHI < 5 events/h), mild OSA (AHI between 5 and 14.9 events/h), moderate OSA (AHI between 15 and 29.9 events/h), and severe OSA (AHI ≥ 30 events/h) [16]. Four oxygen saturation measures were considered: the median and the minimum SpO_2_ level, the cumulative percentage of time spent with oxygen saturations below 90% (CT90), and the 4% oxygen desaturation index (ODI4%).

### 2.5. Excessive Daytime Sleepiness Assessment

Excessive daytime sleepiness was evaluated using the Epworth Sleepiness Scale (ESS), a widely used questionnaire based on one’s likelihood to fall asleep unintentionally during eight daytime situations [17]. A score of 10 or more is considered sleepy. This questionnaire was completed by 80.2% of participants.

### 2.6. Covariates Assessment

Body weight and height were measured without shoes and slight clothing, and body mass index (BMI) was determined from kilograms divided by height in meters squared. Waist and neck circumferences were assessed using a nonstretchable tape with a precision of 0.1 cm. Waist circumference was measured midway between the iliac crest and the lowest rib on the horizontal plane with the individual in a standing position. Neck circumference was assessed in a plane as flat as possible, closely below the laryngeal prominence, while standing erect with eyes facing forward. Blood pressure was assessed in triplicate after five minutes’ rest via an automated device (Omron M6 Comfort HEM-7221-E (Omron Healthcare, Kyoto, Japan)) at 2 min breaks, and the mean of the last two was calculated. Additionally, smoking habits (never, former or current smoker) were also known. Smokers who stopped smoking one or more years prior to visiting were considered former smokers.

### 2.7. Statistical Analysis

As the skewed distribution of all the variables was confirmed by the Shapiro–Wilk test, only nonparametric methods were used. Quantitative data were expressed as median (interquartile range) or as a percentage. The Mann–Whitney U test was used to compare continuous variables, while the Pearson’s Chi-squared test was used to compare categorical records. The relationship between continuous variables was examined by the Spearman correlation test.

Three multiple linear regression models to explore the variables independently related to the AHI, the apnea index and the hypopnea index were used. Variables with a potential impact on sleep breathing function (i.e., age, sex, BMI) and the prediabetes stage were introduced in the models. The adequacy of the regression models was verified through submitting the residuals) to a normality test. All “*p*” values were based on a two-sided test of statistical significance. Significance was recognized at the level of *p* < 0.050. All statistical investigations were completed using the SSPS statistical package (IBM SPSS Statistics for Windows, Version 20.0. Armonk, NY, USA).

## 3. Results

The main clinical characteristics of the study population according to the presence of prediabetes are displayed in Table 1. Subjects with prediabetes were older and presented a higher proportion of women, obesity, and hypertension than control ones.

**Figure 1:**
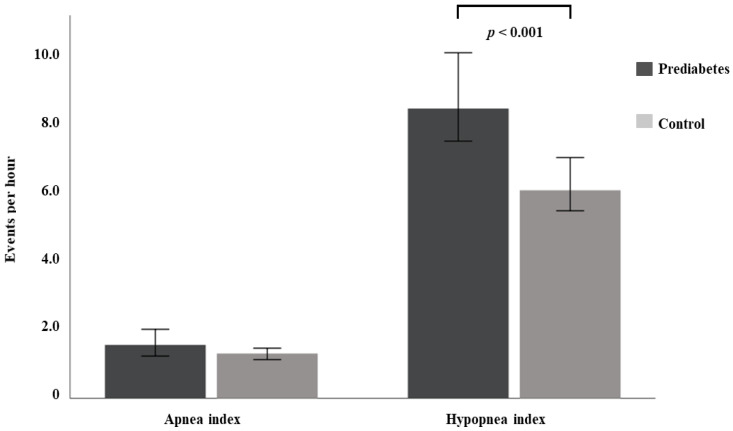
Plot displaying the number of apneas and hypopneas according to the presence of impaired glucose metabolism.

When the sleep breathing was assessed, participants with prediabetes displayed a significantly higher AHI (12.7 (6.1;24.3) vs. 9.5 (4.2;19.6) events/h, *p* < 0.001) than controls (Table 2). The hypopnea index was also greater among participants with prediabetes (8.4 (4.0;14.9) vs. 6.0 (2.7;12.6) events/h, *p* < 0.001), without differences in the apnea index (Figure 1). Altogether, the prevalence of OSA was higher among participants with prediabetes than in controls (78.1 vs. 69.9%, *p* = 0.007). Additionally, patients with prediabetes presented a higher CT90 and ODI4%, as well as lower median and minimum SpO_2_ levels (*p* ≤ 0.001 for all) than patients without prediabetes. No difference in the daytime sleepiness was observed between groups.

Both in the entire population and in those with prediabetes, HbA1c was slightly correlated with the hypopnea index (r = 0.141, *p* < 0.001 and r = 0.171, *p* = 0.002, respectively). HbA1c was also significantly correlated with others nighttime respiratory measurements in both groups (Table 3).

Finally, the multiple linear regression model (Table 4) showed that there was a significant and independent association between the presence of prediabetes and the AHI (B = 2.20 (0. 10 to 4.31), *p* = 0.040) as well as the hypopnea index (B = 1.87 (0. 61 to 3.14), *p* = 0.004), but no with the apnea index.

## 4. Discussion

To the best of our knowledge, this is the first study to provide evidence that prediabetes (after adjusting for age, sex, and presence of obesity) presents with a distinctive sleep breathing pattern, with increased hypopnea episodes but with no differences in apnea events. In our study, and together with classical risk factors for sleep disorders such as age, BMI and male sex, the presence of prediabetes is independently associated with the hypopnea events. These results suggest that the previously evidenced deleterious impact of type 2 diabetes on nocturnal sleep breathing begins in the prediabetes stage, and that any degree of disorder in glucose metabolism will influence this process. Additionally, patients with prediabetes also display a higher prevalence of OSA, as well as an impairment of nocturnal oxygen saturation. These data reinforce and extend previous reports, and run in accordance with the International Diabetes Federation, which recommends screening for OSA subjects with prediabetes [18,19].

Limited information exists on whether prediabetes increases the risk of developing OSA in the general population. A previous cross-sectional study evaluated 137 subjects with extremely obesity who underwent a portable sleep registration at home [19]. According to a standardized 2 h oral glucose tolerance test, the prevalence of OSA increased from 33% in subjects with normal glucose tolerance to 67% and 78% in patients with prediabetes and type 2 diabetes, respectively. Additionally, after adjustment for key clinical and systemic variables such as age, sex, BMI, systemic inflammation, insulin resistance, hypertension, smoking, alcohol consumption and medication, prediabetes was still associated with 3-fold increased odds of OSA compared with those subjects with normal glycaemia [19]. Our study, with a population with a mean BMI of 28.7 kg/m^2^, allows us to analyze the results without the confounding factor of obesity, which directly affects OSA and glucose metabolism abnormalities. However, this study did not provide data related to the number of apneas and hypopneas added together resulting in the AHI. Therefore, our study spread these findings to a broader population with overweight and assessed the components of the AHI to better understand the impact of prediabetes on SBD.

A clear pathophysiology mechanism for the association between glucose abnormalities and sleep breathing has not yet been elucidated. However, insulin resistance, inflammation, visceral adiposity, autonomic dysfunction, and leptin resistance deserve to be commented on. In Sprague Dawley rats, *Ramadan* et al. showed the contribution of insulin resistance to apnea development. Additionally, oral treatment with metformin—an insulin-sensitizer drug—was able to avoid and reverse apnea episodes [20]. Data from 1780 men and 1785 women evaluated within the Epidemiologic Study on the Insulin Resistance Syndrome (DESIR) study showed that insulin resistance was related to a 6-year incident observed apnea during sleep [21]. The standardized odds ratios for fasting plasma insulin, HOMA-IR or triglycerides were 1.31, 1.31, and 1.24, respectively. More interestingly, the relation of insulin resistance and incident observed apnea was homogeneous across BMI classes for both men and women [21]. Similarly, women with polycystic ovary syndrome—characterized by insulin resistance—are more likely to have OSA and experience excessive daytime sleepiness than controls [22]. In addition, insulin resistance may mediate a blockage effect on the pharyngeal dilator muscle, just as the alterations in arterial muscle tone that are well recognized in prediabetes vascular disease [23,24]. Inflammatory processes associated with prediabetes might also affect the upper respiratory tract, reducing the lumen and favoring obstruction [19]. In fact, systemic inflammation measured by fibrinogen and C-reactive protein levels has been associated with nocturnal oxygen saturation parameters and the apnea–hypopnea index in snorers with compromised upper airway anatomy without type 2 diabetes [25]. In addition, other metabolic pathways that would explain the association between prediabetes and OSA have also been suggested, such as microvascular damage, lung microangiopathy, decreased muscle strength, nonenzymatic glycosylation of lung proteins, defects in the bronchiolar surfactant layer and the deficit in glucagon-like peptide 1 concentrations [1].

Our population with prediabetes exhibits a BMI near to 30.0 kg/m^2^, with an increased prevalence of obesity and a higher waist circumference in comparison with control participants. Adiposity may appear as a source of proinflammatory factors as well as lead to the narrowing of the upper airways [26]. However, no differences in neck circumference between groups were observed in our study, making the second option less likely to be responsible for the higher prevalence of OSA in patients with prediabetes. As autonomic dysfunction is already present in prediabetes, both afferent and efferent motor pathway dysfunctions may influence the regulation of blood gases and oxygen delivery, contributing to a blunted ventilatory response to hypoxemia [1,27]. Finally, as leptin has been associated with prediabetes in a dose-dependent manner, pathways related to leptin resistance in type 2 diabetes could also contribute to deficiencies in central respiratory control [28].

As patients with OSA have an almost two-fold higher risk of developing cardiovascular events and all-cause mortality than controls, its increased prevalence could participate in the milieu that favors cardiovascular disease in the prediabetes stage [29,30]. In fact, in a more extensive cohort from the ILERVAS project, subjects with prediabetes presented a higher prevalence of subclinical atheromatous disease than the control group, especially in the carotid territory [31]. Furthermore, nocturnal cycling hypoxia, together with female sex and fasting plasma glucose, has been demonstrated to be independently associated with an increased density of carotid vasa vasorum, an early event in atheromatous disease [32].

Only one study has, so far, focused on weight loss intervention in subjects with both prediabetes and obstructive sleep apnea, showing that changes in SpO_2_ were associated with changes in insulin sensitivity but not with weight loss [33]. Similarly, our group has demonstrated that in type 2 diabetes the improvement of metabolic control achieves a significant reduction in sleep breathing parameters not related with weigh modifications [6]. These results point out a direction for the further improvement of metabolic control in individuals with prediabetes. Whether this optimization must be conducted only with lifestyle changes or medical treatment is not known. For example, glucagon-like peptide (GLP)-1, widely used as anti-diabetic drug, may be an effective therapy for patients with prediabetes and OSA [34,35,36]. Similarly, the combination of metformin and dapagliflozin for 24 weeks in patients with a newly diagnosed type 2 diabetes and OSA achieved a reduction in both the AHI and daily somnolence in comparison with a control group receiving metformin plus glimepriride [37].

Sleep quality, assessed using the Pittsburgh Sleep Quality Index (PSQI), has also been evaluated in subjects with prediabetes [38]. In this way, Iyegha et al. showed how 62% of subjects with prediabetes suffered from poor sleep quality, compared with less than half of normoglycemic subjects [38]. We have no data regarding sleep quality in the ILERVAS project, but no differences in the ESS were observed between participants with and without prediabetes. Our results are in concordance with those of Renko et al., in which daytime sleepiness was not linked with using sleep medication or impaired glucose regulation [39]. Altogether, this may suggest that sleep quality is more sensitive to the prediabetes stage than daytime sleepiness.

This study has a few limitations that need to be addressed. Currently, we have diagnosed prediabetes according only to HbA1c values, one of the three possibilities accepted by the American Diabetes Association. However, as different tools identify different populations, future studies using fasting plasma glucose (impaired fasting glucose) and 2 h plasma glucose (impaired glucose tolerance) are needed. Second, we have no information about the time of appearance of prediabetes in our population, a factor that might influence our results similarly to how the known evolution time in type 2 diabetes affects the incidence of classical chronic complications. Third, the gold standard diagnosis of OSA according to the American Academy of Sleep Medicine is through polysomnograms, not cardiorespiratory polygraphs. The latter shows a lower diagnostic performance compared to polysomnography that is related to the difficulty in identifying apneas and especially hypopneas. However, population-based studies assessing sleep breathing parameters in large populations are easily performed with at-home registers. In addition, this fact amplifies the significance of our results, in which a higher prevalence of hypopnea events in patients with prediabetes compared to the control group has been detected. Fourth, we have no circulating biomarkers of insulin resistance or systemic inflammation, and therefore, their potential role in this association could not be assessed in our study. Finally, Fifth, we do not have data on psychiatric illnesses in our population, which have a direct effect on the severity of OSA. Moreover, the cross-sectional nature of the study does not allow us to establish causality with the results, as well as characteristic of the ILERVAS population (Spanish middle-aged individuals with low-to-moderate cardiovascular risk) precludes us from generalizing our results to the global population. Finally, future studies need to be designed to better evaluate the underlying mechanisms that link prediabetes with OSA and its clinical significance.

## 5. Conclusions

In summary, the prevalence of OSA was significantly higher in participants with prediabetes that in the control group in a large cohort study of Spanish subjects with low-to-moderate CV risk. After adjusting for age, sex, and the presence of obesity, the increased AHI in participants with prediabetes was mainly associated with increments in the hypopnea events, supporting the hypothesis that glucose abnormalities exert a lineal negative impact on SBD, from insulin resistance with normal fasting glucose to confirmed diagnosis of type 2 diabetes. Moreover, the presence of prediabetes independently predicted the AHI and the hypopnea events per hour. Additional studies to identify subjects with prediabetes more vulnerable to experiencing problems with nighttime respiratory function, and factors that accelerate its progression and severity, are needed.

## Figures and Tables

**Table 1:** Main clinical characteristics of the study population according to the presence of prediabetes.

	Prediabetes (*n* = 311)	Control Group (*n* = 655)	*p*
Age (years)	59 (54;63)	56 (52;61)	<0.001
Women, *n* (%)	180 (57.8)	316 (48.2)	0.004
HbA1c (mmol/mol)	40 (39;42)	36 (33;37)	<0.001
HbA1c (%)	5.8 (5.8;6.0)	5.4 (5.2;5.5)	<0.001
Hypertension, *n* (%)	147 (47.2)	240 (36.6)	0.001
Systolic blood pressure (mm Hg)	132 (120;143)	128 (118;139)	0.008
Diastolic blood pressure (mm Hg)	81 (75;88)	81 (74;87)	0.569
Obesity, *n* (%)	124 (39.8)	177 (27.0)	<0.001
BMI (Kg/m^2^)	29.7 (26.9;33.2)	27.9 (24.9;31.0)	<0.001
Waist circumference (cm)	103 (96;110)	98 (92;106)	<0.001
Neck circumference (cm)	37.5 (35.0;41.0)	37.5 (34.5;41.0)	0.261
Current or former smoker, *n* (%)	184 (59.1)	430 (65.6)	0.050

Information is displayed as a median (interquartile range) or *n* (percentage). HbA1c: glycated hemoglobin; BMI: body mass index.

**Table 2:** Nighttime respiratory characteristics of the study population according to the presence of prediabetes.

	Prediabetes (*n* = 311)	Control Group (*n* = 655)	*p*
Time of evaluation (hs)	7.2 (6.4;8.0)	7.2 (6.5;8.0)	0.684
AHI (events/h)	12.7 (6.1;24.3)	9.5 (4.2;19.6)	<0.001
Apnea index (events/h)	1.6 (0.4;5.8)	1.3 (0.4;4.3)	0.159
Hypopnea index (events/h)	8.4 (4.0;14.9)	6.0 (2.7;12.6)	<0.001
OSA, *n* (%)	243 (78.1)	458 (69.9)	0.007
Mild OSA, *n* (%)	108 (34.8)	227 (34.6)	0.142
Moderate OSA, *n* (%)	76 (24.4)	138 (21.0)	0.060
Severe OSA, *n* (%)	57 (18.5)	89 (13.6)	0.010
Median SpO_2_ level (%)	92 (91;93)	93 (92;94)	<0.001
Minimum SpO_2_ level (%)	82 (77;85)	83 (80;87)	<0.001
CT90 (%)	14 (4;33)	6 (1;24)	<0.001
ODI4% (events/h)	14 (8;27)	11 (5;21)	0.001
Epworth Sleepiness Scale score *	4 (2;5)	3 (2;5)	0.740

Information is displayed as a median (interquartile range) or *n* (percentage). AHI: Apnea–hypopnea index; OSA: obstructive sleep apnea; SpO_2_: oxygen saturation; CT90: cumulative time percentage with SpO_2_ < 90%; ODI4%: number of 4% oxygen desaturation index; * The Epworth Sleepiness Scale was completed by 80.2% of participants.

**Table 3:** Correlations of sleep respiratory measurements with glycated hemoglobin in the entire population and in participants with prediabetes.

	Prediabetes (*n* = 311)		Entire Population (*n* = 966)	
	r	*p*	r	*p*
AHI (events/h)	0.093	0.099	0.131	<0.001
Apnea index (events/h)	−0.039	0.492	0.065	0.042
Hypopnea index (events/h)	0.171	0.002	0.141	<0.001
Median SpO_2_ level (%)	−0.166	0.003	−0.204	<0.001
Minimum SpO_2_ level (%)	−0.126	0.025	−0.159	<0.001
CT90 (%)	0.144	0.010	0.209	<0.001
ODI4% (events/h)	0.123	0.028	0.150	<0.001
Epworth Sleepiness Scale score *	0.009	0.884	−0.035	0.322

AHI: Apnea–hypopnea index; SpO_2_: oxygen saturation; CT90: cumulative time percentage with SpO_2_ < 90%; ODI4%: number of 4% oxygen desaturation index; * The Epworth Sleepiness Scale was completed by 80.2% of participants.

**Table 4:** The multiple linear regression model for the AHI, apnea index and hypopnea index.

AHI (Events/h) R^2^ = 0.12	B (95% IC)	Standardized Regression Coefficients	*p*
Age (Years)	0.36 (0.19 to 0.52)	0.14	<0.001
Sex (Male)	8.29 (6.23 to 10.35)	0.24	<0.001
Obesity (BMI ≥ 30 kg/m^2^)	8.54 (6.44 to 10.64)	0.26	<0.001
Prediabetes (HbA1c 5.7 to 6.4%)	2.20 (0. 10 to 4.31)	0.58	0.040
Apnea index (events/h) R^2^ = 0.06			
Age (Years)	0.21 (0.13 to 0.30)	0.17	<0.001
Sex (Male)	3.58 (2.55 to 4.62)	0.20	<0.001
Obesity (BMI ≥ 30 kg/m^2^)	1.73 (0.67 to 2.78)	0.11	0.001
Prediabetes (HbA1c 5.7 to 6.4%)	0.16 (−0. 90 to 1.22)	0.00	0.765
Hypopnea index (events/h) R^2^ = 0.12			
Age (Years)	0.13 (0.03 to 0.23)	0.11	0.013
Sex (Male)	3.81 (2.57 to 5.05)	0.15	<0.001
Obesity (BMI ≥ 30 kg/m^2^)	5.94 (4.68 to 7.21)	0.29	<0.001
Prediabetes (HbA1c 5.7 to 6.4%)	1.87 (0. 61 to 3.14)	0.82	0.004

B: unstandardized beta; AHI: Apnea–hypopnea index; BMI: body mass index. The adequacy of the regression models was verified through submitting the residuals to a normality test: in all cases *p* < 0.001.

## Data Availability

The data presented in this study are available on request from the corresponding author. The data are not publicly available due to the signed consent agreements around data sharing, which only allow access to the researchers of the study following the project purposes. Requestors wishing to access the data used in this study can make a request to M.B.-L. The request will be subjected to approval and formal agreements regarding confidentiality and secure data storage being signed the data would be the provided.

## Supplementary Materials

The following supporting information can be downloaded at: https://www.mdpi.com/article/10.3390/jcm11051413/s1, Figure S1: Flow chart in study population.
