# Supplementary material for: Prediabetes Is Associated with Increased Prevalence of Sleep-Disordered Breathing
Source: J Clin Med. 2022 Mar 4;11(5):1413. doi: 10.3390/jcm11051413 (PMC8910907; doi:10.3390/jcm11051413)
Supplement: Supplementary file 1 [file jcm-11-01413-s001.zip › jcm-1594860-supplementary.pdf]

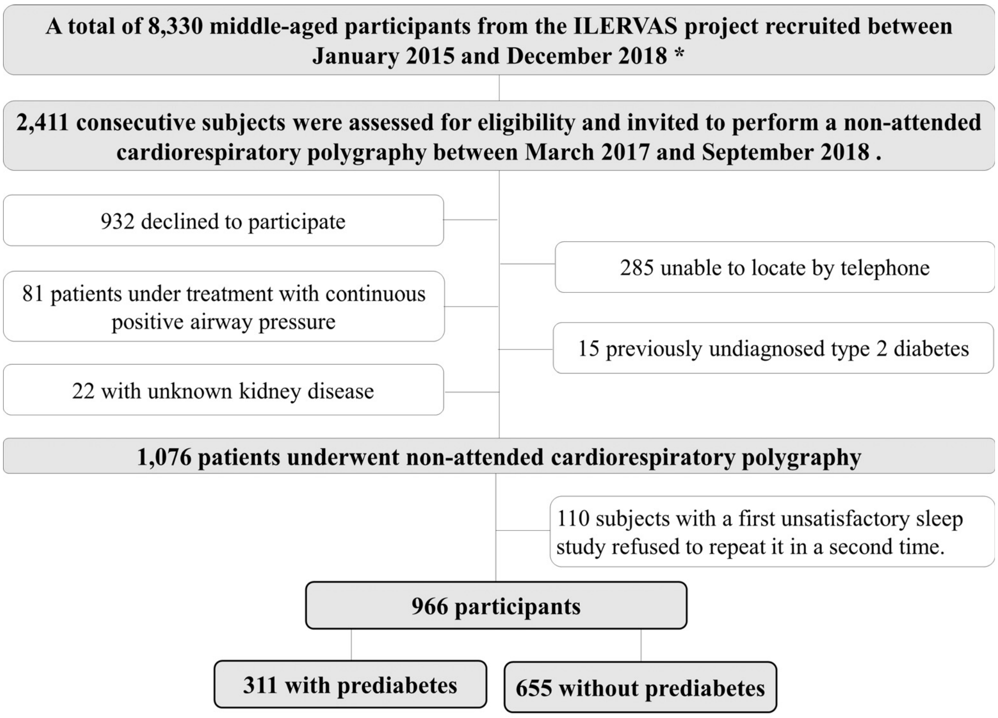

* The inclusion criteria were women aged between 50 and 70, men aged between 45 and 65, and presence of at least one cardiovascular risk factor (dyslipidemia, hypertension, obesity, smoking and/or having a first degree relative with premature cardiovascular disease). The exclusion criteria were medical history of cardiovascular disease, type 2 diabetes, chronic kidney disease, active neoplasia, a life expectancy less than 18 months, institutionalized population and pregnancy.

Figure S1. Flow chart in study population.
